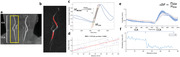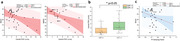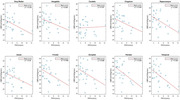# Carotid stiffening with impaired damping is associated with cognitive impairment and reduced cerebral perfusion in elderly adults

**DOI:** 10.1002/alz.093187

**Published:** 2025-01-09

**Authors:** Jianing Tang, Tianrui Zhao, Elizabeth B Joe, Soroush Heidari Pahlavian, Helena C Chui, Lirong Yan

**Affiliations:** ^1^ Department of Radiology, Northwestern University, Chicago, IL USA; ^2^ Department of Neurology, Keck School of Medicine, University of Southern California, Los Angeles, CA USA; ^3^ Stevens Neuroimaging and Informatics Institute, University of Southern California, Los Angeles, CA USA

## Abstract

**Background:**

Emerging evidence indicates that arterial stiffening is associated with aging and cognitive impairment. Arterial stiffness is typically assessed by measuring pulse wave velocity (PWV) between carotid and femoral arteries. A recent study has introduced a fast oblique‐sagittal PC‐MRI (OS PC‐MRI) technique that allows for simultaneously quantifying carotid PWV (cPWV) and CCA‐ICA damping factor (cDF) within 2 minutes. This study aims to investigate the relationship between the pulsatile measures by OS PC‐MRI and cognitive impairment and cerebral perfusion in elderly adults.

**Method:**

Forty elderly participants (22 female,73.3 ± 7.7 years) were enrolled in the study and underwent MRI scans at 3T including a vessel scout, retrospectively gated OS PC‐MRI (n=40), and pCASL (n=25). Neuropsychological tests were also conducted, including Clinical Dementia Rating (CDR, n=29), Mini‐Mental State Exam (MMSE, n=29), and Montreal Cognitive Assessment (MoCA, n=40). As shown in Figure 1, in OS PC‐MRI, Pearson correlation was performed to test the associations of the metrics pulsatile measures with cognitive functions and cerebral perfusion.

**Result:**

Elevated cPWV was strongly associated with cognitive decline after controlling for age, gender, and years of education (cPWV vs. MoCA: r = ‐0.36, p = 0.03; cPWV vs. MMSE: r = ‐0.53, p = 0.005;) (Figure 2a). The participants with CDR>0 (n=19) showed higher cPWV values compared to those with CDR=0 (p=0.0045) (Figure 2b). cDF showed a significant correlation with MoCA (p = 0.036) (Figure 2c). Increased cPWV was also associated with downstream CBF in gray matter (p=0.034) and AD‐related regions (Figure 3).

**Conclusion:**

Our results showed that both elevated CCA‐ICA PWV and DF were strongly associated with cognitive impairment and downstream cerebral perfusion deficits in elderly adults. These findings suggest that arterial stiffening with reduced damping leads to excessive transmission of pulse energy to downstream vasculature resulting in microvascular dysfunction and thus cognitive impairment.